# Speed and Shape of Population Fronts with Density-Dependent Diffusion

**DOI:** 10.1007/s11538-024-01381-2

**Published:** 2024-11-09

**Authors:** Beth M. Stokes, Tim Rogers, Richard James

**Affiliations:** 1 Department of Mathematical Sciences, University of Bath, Bath, UK; 2 Department of Physics, University of Bath, Bath, UK

**Keywords:** Density dependent diffusion, Travelling waves

## Abstract

There is growing empirical evidence that animal movement patterns depend on population density. We investigate travelling wave solutions in reaction-diffusion models of animal range expansion in the case that population diffusion is density-dependent. We find that the speed of the selected wave depends critically on the strength of diffusion at low density. For sufficiently large low-density diffusion, the wave propagates at a speed predicted by a simple linear analysis. For small or zero low-density diffusion, the linear analysis is not sufficient, but a variational approach yields exact or approximate expressions for the speed and shape of population fronts.

## Introduction

There has been an upturn of interest in recent years in the study of animal movement, spanning the range of scales from a few interacting individuals to movement of entire populations. Much of this increase, and the emergence of movement ecology as a field of study (Nathan et al. 2008), has been fueled by technological advances that enable not only the tracking or biologging of individual animals (Krause et al. (2013), see Gottwald et al. (2019); Kazimierski et al. (2023) for some recent examples) but also the ability to share raw tracking data with the wider scientific community and the public (Kays et al. 2022); armchair ecologists can now routinely follow the movements of migrating birds and mammals on their phones. There is also considerable interest in ecologically and economically important population-level movement such as the invasion of novel species into new territory, or population range expansion in response to changing environmental conditions. Empirical studies have begun to explore the importance of the density of conspecifics in enhancing or suppressing animal movement, see for example, Jreidini and Green (2024) and references therein. We now seek theoretical insights into this question.

Reaction-diffusion PDEs remain a useful, if often over-simplified, route to modelling large-scale movement of populations into new territory, giving rise to constant-form travelling waves. The “reaction term” (actually population growth) is assumed to be density-dependent, but much less attention has been given to the possibility that the diffusion term, representing animal movement, is density-dependent. The aim of this paper is to explore the effect of density-dependent diffusion on the existence, speed and shape of travelling wave solution to PDE models of population invasion.

The starting point for describing the rate of change of density $u(x,t)\in [0,1]$ with time $t$ in one spatial dimension $x$ is the Fisher-Kolmogorov-Petrovskii-Piskunov (FKPP) equation (Fisher 1937; Kolmogorov et al. 1937)

1 $$\begin{aligned} \frac{\partial u}{\partial t} = D\frac{\partial ^2 u}{\partial x^2} + f(u), \end{aligned}$$

with diffusion described by the (constant) coefficient *D*. The growth term *f*(*u*), with $f(0)=f(1)=0$ is most frequently chosen to be of the logistic form $f(u)=ru(1-u)$ with *r* a positive parameter fixing the rate of growth. The FKPP equation admits travelling wave solutions $u(x,t)=u(x-ct)$, moving at constant speed *c* and of fixed profile. It is this feature which makes (1) a natural starting point for many models of, among other things, the expansion of animal population fronts into new territory (eg. Murray (2002)).

If the growth *f*(*u*) is logistic, and *D* a constant, the travelling wave solutions are of the ‘pulled’ type (Van Saarloos 2003); the wavefront *u*(*x*, *t*) is smooth for small *u*, and the speed of propagation is determined by a linear analysis around the unstable state $u=0$. In brief, setting $u(x,t) = u(z)$ with $z=x-ct$ in (1) gives $Du''+cu'+f(u)=0,$ which admits travelling wave solutions for all speeds $c \ge c_{L}=2\sqrt{f'(0)D}$.

Which of the possible wavespeeds is *selected* is less straightforward to determine. Numerical solutions of (1) with $f(u)=ru(1-u)$ and with step-function initial conditions ($u(x,0)=1$ if $x\le x_0$, 0 otherwise) will always, modulo numerical error, yield wavefronts moving with speed $c_{L}=2\sqrt{rD}$. This is as expected; if initial conditions decay faster than a sufficiently steep exponential, the waves have a universal relaxation behaviour to the minimum physically realisable speed as $t \rightarrow \infty$ (Van Saarloos and Ebert 2000). (This condition is itself a relaxation of the one originally given by Kolmogorov et al. (1937), that initial conditions with compact support will lead to selection of $c_L$.) Furthermore, any numerical solution of (1) will necessarily be performed on a finite domain, and all solutions with speed greater than the minimum will be unstable to fluctuations in the moving frame of the wave (Canosa 1973). So, with some care given to the choice of initial conditions, the solution of the FKPP equation will give rise to travelling waves moving at the *minimum realisable speed* $c_{L}=2\sqrt{rD}$. Note that although the speed is known exactly, there is no simple formula for the wave profile *u*(*z*).

Many extensions to analysis of the FKPP equation (1) have explored the effect on population dynamics of a range of growth (or reaction) functions *f*(*u*). Some of the corresponding PDEs are exactly solvable; an example to which we return has cubic growth $f(u)=u(1-u)(u-a)$, $a\in (0,1/2)$. This case is often referred to as the Nagumo equation, seemingly with reference to a model of neurons (Nagumo et al. 1962). Most of the key features of the linear analysis above carry over to general *f*(*u*) (Murray 2002), providing *f*(*u*) has only two zeros, $u_1, u_2$ with $u_1 < u_2$; $f'(u_1) > 0$ and $f'(u_2) < 0$. Usually, as here, $u_1 = 0$, $u_2 = 1$.

There has been less attention given to the existence and properties of travelling wavefronts when diffusion is density-dependent. Considering density-dependent *D*(*u*) will allow us to model behavioural aspects of the dispersal of animal populations into new territory and explore the effect of these behaviours on the corresponding travelling wave solutions. There are many reasons for individuals and populations to undertake movements, such as searching for food or breeding sites, avoiding inbreeding, and in response to man-made factors such as deforestation or climate change. Ecological theorists have predicted that individuals should disperse away from areas of high density and high competition if they can expect to settle in areas of low density and low competition (Poethke and Hovestadt 2002; Travis et al. 1999); in other words, animals move more at higher density to avoid overcrowding. Many animal populations, from invasive crayfish (Galib et al. 2022) to meerkats (Maag et al. 2018) and black bears (Kopsala et al. 2023), have been found to exhibit some form of positive density-dependent dispersal, but this is not always the case. Negative density-dependent dispersal has been observed in various populations, including microorganisms (Jacob et al. 2016), insects (De Meeus et al. 2019), fish (De Bona et al. 2019) and mammals (Fattebert et al. 2015). Proposed mechanisms for this effect can be broadly categorised as either passive effects due to population structure (Matthysen 2005), or attractive forces which hold back range expansion (Bowler and Benton 2005; Travis et al. 1999).

Density-dependent diffusion *D*(*u*) can be included in the FKPP equation as

2 $$\begin{aligned} \frac{\partial u}{\partial t} = \frac{\partial }{\partial x} \left[ D(u) \frac{\partial u}{\partial x} \right] + f(u). \end{aligned}$$

This PDE has been analysed for relatively few choices of diffusion function. It has been analysed in detail for functions of the form $D(u) = u^m$. The case $m < 0$ models negative density-dependent diffusion, sometimes referred to as ‘fast diffusion’. King et al. (2003) investigate the problem for a range of $m < 0$. They derive approximate analytical solutions, investigate the asymptotic behaviour in both the limits $t \rightarrow \infty$ and $m \rightarrow 0^-$ and present numerical simulations. They find that this form of negative density-dependent diffusion leads to *accelerating* wavefronts, not permanent-form travelling wave solutions.

The case $D(u)=u$ (i.e. $m = 1$), exhibiting positive density-dependence, is exactly solvable (Aronson 1980; Newman 1980). With $f=u(1-u$) it yields a travelling wave moving at the unique speed $c = 1/\sqrt{2}$ and with a profile $u(z)=\max \{0,1-\frac{1}{2}\exp (z/\sqrt{2})\}$ - see Fig. 3. This case is clearly not amenable to simple linear stability analysis; *u*(*z*) is a *sharp-fronted* travelling wave with discontinuous slope where the density hits $u=0$, and no smooth small-*u* lead at the front of the wave. Sánchez-Garduño and Maini (1994, 1995, 1997) have looked in detail at sharp-fronted travelling wave solutions for different forms of reaction-diffusion equation. In Sánchez-Garduño and Maini (1994) they show that ‘degenerate’ diffusion functions with $D(0) = 0$ and $D(u) > 0$ for all $u \in (0,1]$ are guaranteed (for appropriate *f*(*u*)) to produce a sharp-fronted travelling wave. Malaguti and Marcelli (2003) show that degenerate ($D(0)=0$) and doubly-degenerate (*D*(1) also zero) diffusion always leads to a sharp-fronted wave travelling at some unique speed $c^*$ ($=1/\sqrt{2}$ for $D=u$) and to a continuum of smooth waves at faster speeds. Sherratt (2010) uses singular perturbation theory to find an asymptotic approximation to wave solutions travelling faster than $c^*$.

The aim of this paper is to explore the effect of a wider range of diffusion density dependence on travelling wave solutions to (2). Using analytical means where possible, and supported by simple finite-difference numerical simulations, we derive results for the selected speed *c* of travelling waves, and explore the effect of *D*(*u*) on the wave profile *u*(*z*). In the next section we give an overview of linear and variational methods (following Hadeler (1983) and Benguria and Depassier (1996)) for computing wave speed in the density-dependent FKPP equation. The remainder of the paper is then arranged around three case studies, designed to illustrate different classes of behaviour for the selected wavespeed and front shape. Each case study employs a different family of diffusion function *D*(*u*). We have deliberately chosen these functions to be as simple as possible (*D* is always continuous and piecewise linear) to represent both positive- and negative-density dependence, and regard them as stylised representations of the sorts of variation of diffusion with density that might be expected in animal populations.

## Methods

We seek solutions to the FKPP equation with density-dependent diffusion (2). In all cases, *D*(*u*) is non-negative for all $u \in [0,1]$. For the most part we will restrict attention to logistic growth $f=u(1-u)$ (with rate $r=1$), though some of the results we obtain hold for more general growth functions, subject to simple constraints.

### Linear Analysis

For some, but not all, diffusion functions *D*(*u*) a simple linear stability analysis produces a lower bound $c_L$ on wavespeed, and numerical solutions of (2) select waves travelling at $c_L$, as for the FKPP equation (1). Linearisation of (2) proceeds as follows: set $z=x-ct$ to produce the ODE for *u*(*z*)

3 $$\begin{aligned} D(u)\frac{\text {d}^2 u}{\text {d}z^2} + \frac{\text {d} D}{\text {d} u} \left( \frac{\text {d} u}{\text {d} z}\right) ^2 +c\frac{\text {d} u}{\text {d} z} + f(u)=0. \end{aligned}$$

Let $v = - \text {d} u/\text {d} z$ so that (3) can be written as the coupled ODE system

4 $$\begin{aligned} \frac{\text {d} u}{\text {d} z} = -v, \quad \frac{\text {d} v}{\text {d} z} = \frac{v^2 D'(u) - cv + f(u)}{D(u)}, \end{aligned}$$

where $D'(u)$ indicates a derivative with respect to *u*. The system (4) is the starting point for a linear stability analysis of the problem, looking at critical points of trajectories in the (*u*, *v*) phase plane; for our choice of variables, trajectories are all in the quadrant where *u* and *v* are both non-negative.

The variable $z=x-ct$ traverses a right-moving wave from left to right, therefore we seek the heteroclinic orbit departing $(u,v)=(1,0)$ and arriving at $(u,v)=(0,0)$. In the neighbourhood of zero, with $D(0) \ne 0$, the system (4) linearises as

5 $$\begin{aligned} \frac{\text {d}}{\text {d} z}\begin{pmatrix}u\\ v\end{pmatrix}=\frac{1}{D(0)}\begin{pmatrix}0& -D(0)\\ f'(0)& -c\end{pmatrix}\begin{pmatrix}u\\ v\end{pmatrix}\,. \end{aligned}$$

Depending on the value of *c*, the eigenvalues of this linear system may or may not be real, but complex eigenvalues would imply oscillatory dynamics that are not physically realisable as we do not permit negative population density. It is straightforward to compute the necessary condition that the speed *c* must exceed $c_L:=2\sqrt{f'(0)D(0)}$. That is, all travelling wave solutions to (2) must have speed at least as fast as the *linear speed* $c_L$. For some choices of *D*(*u*), however, it is possible that this speed is not physically realisable, by which we mean there exists a stationary positive $u(z) \in [0,1]$ that solves the PDE and moves with speed *c*. In this case the selected wavefront will in fact travel faster and alternative methods will be needed to calculate the speed.

**Fig. 1:**
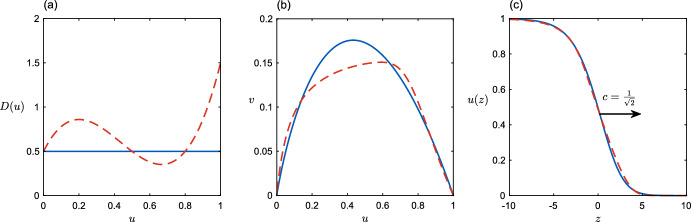
An illustration of solutions to (2) for two choices of diffusion function *D*(*u*) (**a**), one constant (solid line) and one with an arbitrarily-shaped density dependence but with *D*(0) not too small (dashed line). The corresponding (*u*, *v*) phase plane trajectories (**b**) are clearly quite different, but the resulting wave profiles (**c**) are almost identical. The selected speed for each wave is the linear speed $c_L=1/\sqrt{2}$ in this case (Color figure online)

Figure 1c shows travelling waves for two diffusion functions that have the same $D(0)=0.5$. They travel at the same speed $c_L=1/\sqrt{2}$ and have almost identical wave profiles *u*(*z*). We find much the same outcome for more-or-less any diffusion function *D*(*u*) with *D*(0) finite and not too small; more examples are found in Sect. 3.1. For cases where *D*(0) is small, a different approach is needed.

### Variational Principle

Monotonicity of the wave front implies that we can write *v* as a function of *u*, thereby eliminating explicit dependence on *z* from the system (4) to leave

6 $$\begin{aligned} v \frac{\text {d}}{\text {d} u} \left[ D(u) v\right] - cv + f(u) = 0. \end{aligned}$$

This ODE for *v*(*u*) will be the starting point for all our subsequent analysis of the speed and shape of travelling wave solutions of (2). Unless stated otherwise, $f(u)=u(1-u).$

To derive the variational principle we will closely follow the arguments in Benguria and Depassier (1996). For the most part they deal with the case of constant diffusion, paying more attention to general growth functions *f*(*u*). Though they do not explicitly write down exactly the scheme we use, the key result we need appears in Benguria and Depassier (1996) and, in a slightly different form in Hadeler (1983).

Let *s*(*u*) be any monotonically increasing function with $s(0) = 0$ and $s \rightarrow \infty$ as $u \rightarrow 1$. Multiplying (6) by $\frac{D(u)}{s(u)}$ and integrating by parts with respect to *u* we obtain

7 $$\begin{aligned} \int _0^1 \frac{f D}{s} du = c \int _0^1 \frac{D v}{s} du - \frac{1}{2} \int _0^1 \frac{s'}{s^2} (Dv)^2 du. \end{aligned}$$

Our choice of *s*(*u*) means all the integrands in (7) are non-negative, so it is useful to define the function

8 $$\begin{aligned} \phi (v) = c\frac{Dv}{s} - \frac{1}{2} \frac{s'}{s^2} (Dv)^2\,. \end{aligned}$$

For fixed *u*, assuming $D(u)>0$, $\phi$ achieves a strictly positive maximum at

9 $$\begin{aligned} v_{max} = c\frac{s}{s' D}. \end{aligned}$$

It follows that for all *u* (including when $D(u)=0$),

10 $$\begin{aligned} \phi (v) \le \frac{c^2}{2s'}, \end{aligned}$$

and substituting (10) into the integrands on the RHS of (7), we have that

11 $$\begin{aligned} c^2 \ge 2 \frac{\int _0^1 (fD / s)du}{\int _0^1 (1 / s') du}. \end{aligned}$$

This (for a different choice of trial function) is the bound given in Benguria and Depassier (1996) for density-dependent diffusion, as an aside to their main result, which considered constant diffusion. Taking the supremum over all trial functions *s*(*u*) for which the integrals exist, the maximisation principle is

12 $$\begin{aligned} \frac{1}{2} c^2 = \sup _{s} \frac{\int _0^1 (fD / s)du}{\int _0^1 (1 / s') du}. \end{aligned}$$

This is a slightly unusual route to a variational scheme, in that the trial functions *s*(*u*) are not trial solutions to the ODE (6) for *v*(*u*). We therefore need also to show that there exists some $\hat{s}(u)$ for which the equality in (12) holds. Then if we can find $\hat{s}(u)$, (12) enables us to find the *exact* wavespeed. In cases where $D(u)>0$ for all *u*, from (9), we know that any such $\hat{s}(u)$ will satisfy

13 $$\begin{aligned} \frac{\text {d} \hat{s}}{\text {d} u} = \frac{c \hat{s}}{Dv}, \end{aligned}$$

which can be integrated to obtain (up to a constant)

14 $$\begin{aligned} \hat{s}(u) = \exp \left( \pm c \int \frac{du}{Dv} \right) . \end{aligned}$$

Equations (12) and (14) describe a self-consistent scheme relating the wave speed to the maximising trial function. In most cases, however, the appearance of *c* as a parameter in (13) spoils any hope of obtaining $\hat{s}$ as a precursor to computing the wave speed.

If $\hat{s}$ is not known then there are two main routes to proceed: find an approximation to $\hat{s}$, or select a parameterised family of functions to focus on regions of interest in the integral of *fD*/*s*. An instructive example is the family

15 $$\begin{aligned} s(u)=\left( \frac{u}{1-u}\right) ^\beta \,, \end{aligned}$$

in which the parameter $\beta$ enables control over the pole at zero. In fact, the linear speed $c_L$ is recovered as the estimate obtained as $\beta$ approaches its upper limit. In the most simple case that $f'(0)$ and *D*(0) are both positive and finite, the calculation proceeds as follows. In the limit $\beta \nearrow 2$, the function $(u/(1-u))^\beta$ is proportional to a Dirac delta centred at zero, so the leftmost limit of the integral involving *f* and *D* will dominate. We compute

$$ \frac{1}{2}c^2=\sup _{s} \frac{\int _0^1 (fD / s)du}{\int _0^1 (1 / s') du}\ge \lim _{\beta \nearrow 2}\frac{\beta \int _0^1 fDu^{-\beta }(1-u)^{\beta }du}{\int _0^1 u^{1-\beta }(1-u)^{1+\beta } du}=\frac{1}{2}c_L^2\,, $$

where the integral was performed in the limit by expanding $f(u)D(u)=uf'(0)D(0)+\mathcal {O}(u^2)$. If either *f* or *D* has a different behaviour near zero (for example growing like some other power of *u*) the upper limit for $\beta$ will be different. This restriction holds more generally since any choice of *s* must be sufficiently fast growing near zero so that the singularity in *fD*/*s* is integrable. More interestingly, it is not *a priori* clear when the supremum over $\beta$ will be obtained at an endpoint – we will see a case later where it is not.

Every variational principle has associated with it an Euler-Lagrange equation. For (12) it would be expected that this would be an ODE for *s*(*u*), but re-arranging for *u*(*s*) yields a more natural form for the associated Euler-Lagrange equation, which becomes

16 $$\begin{aligned} c^2 \frac{\text {d}^2 u}{\text {d} s^2} + \frac{f D}{s^2} = 0. \end{aligned}$$

We now have three ordinary differential equations ((6), (13) and (16)) all of which offer routes to calculating wavespeed *c*. None of them gives a general route to finding the wave profile; even when $\hat{s}(u)$ is known, there is no guarantee that (14) can be inverted to find *u*(*z*). Note that when $D=1$, (14) gives $\hat{s}(u) = \exp (-cz)$, exactly the change of variable needed to transform (6) into (16) in that case (Benguria and Depassier 1996).

One of the potential attractions of this variational approach is that *f*(*u*) and *D*(*u*) appear in the same term, as a simple product (compare (16) and (11) with (6)). Therefore, known results for the FKPP equation (1) with *constant* diffusion and a growth function *f*(*u*)*D*(*u*) can in principle be exploited to analyse at least the wavespeed of solutions to the density-dependent FKPP equation (2) with diffusion *D*(*u*) and growth *f*(*u*). This fact was recognised and exploited by Hadeler (1983), and developed further by Malaguti and Marcelli (2002). We make use of this feature in Sect. 3.2.

### Numerical Simulations

For most choices of growth *f*(*u*) and diffusion *D*(*u*) there is no analytic expression for the wave profile *u*(*z*), so in all case studies we simulate solutions to the PDE (2) to generate *u*(*z*) and to compare observed speeds to theory. The simulations adopt a simple finite difference scheme on regular grids in time (interval $\delta t$) and space ($\delta x$). We take the first discrete spatial derivative in (2) to the right, and the second to the left. At an interior point on the space grid $x_i$, the density $u_i:=u(x_i)$ is therefore updated each timestep according to

17 $$\begin{aligned} u_i\rightarrow u_i+Q\left[ D(u_i) (u_{i+1}-u_i) - D(u_{i-1})(u_i-u_{i-1})\right] +\delta t f (u_i)\,, \end{aligned}$$

where $Q=\delta t/(\delta x)^2$. For the simplest case that *D* is constant, the scheme is a straighforward “Forward Time, Centred Space” discretisation, for which $Q<1$ assures numerical stability. We chose $Q=0.2$.

**Fig. 2:**
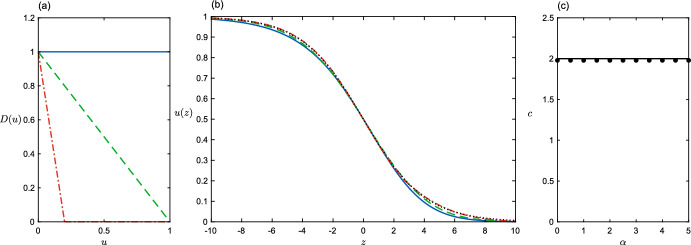
Negative density-dependent diffusion $D(u)= \max \{0, 1-\alpha u\}$ (**a**). The solid line $\alpha =0$ has constant diffusion, for comparison. The other cases in (**a**) and (**b**) are $\alpha =1$ (dashed line) and $\alpha =5$ (dot-dashed). The corresponding wave profiles *u*(*z*) for these three cases are shown in (**b**), as is the analytic result for $\alpha \rightarrow \infty$ (dotted line). (**c**) Wavespeed as a function of $\alpha$ - dots are from simulation, solid line is the linear speed $c_L$ (Color figure online)

In every simulation we used the initial condition $u(x,0)=\min \{1,\max \{0,1-x/W\}\}$ (we chose $W=10$), and the simulations have a transient period $T\gg \delta t$ before the profile is recorded and centred on $z=0, u=0.5$ for presentation. The wavespeed is calculated from the $x-$coordinate of the wave with $u=0.5$ at times *T* and 2*T*. The difference between the predicted and measured wavespeeds (in Fig. 2c for example) are within the expected discretisation error of our numerical scheme (Van Saarloos 2003).

## Case Studies

### Negative Density Dependence

In general, for sufficiently steep initial conditions (Kolmogorov et al. 1937; Van Saarloos and Ebert 2000), diffusion functions with *D*(0) sufficiently large will lead to a pulled wave propagating at the linear speed $c_L=2\sqrt{f'(0)D(0)}.$ This is illustrated in Fig. 1, and again in Fig. 2 for the simple family of functions $D(u) = \max \{0, 1- \alpha u\}, \alpha > 0$. These functions constitute our first case study, exhibiting negative density-dependent diffusion, which has been observed in a wide variety of taxa (Fattebert et al. 2015; Jacob et al. 2016; De Bona et al. 2019). The growth function is $f=u(1-u)$, so $c_L=2.$

For this set of diffusion functions the shape of the wavefront can be determined exactly in the population bulk, i.e. the region $u>1/\alpha$, when $\alpha >1$. Inserting $c=2$ and $D=0$ in (6) and solving for *v* and then *u* via the definition $\frac{du}{dz} = -v$, we obtain $u(z)=\frac{1}{2}(1-\tanh (z/4))$ for $z\in (-\infty ,4\tanh ^{-1}(1-2/\alpha )]$. Note that this result corresponds exactly to the solution of the logistic growth model; looking back into the population bulk is equivalent to looking forward in time at logistic growth. In the low-population tail of the wave, (6) does not admit an exact solution. Interestingly, expansion around the endpoint $u\nearrow 1/\alpha$ yields non-singular behaviour in *v* only when $v\rightarrow \alpha (\sqrt{2-\alpha }-1)$, which is different from the limit $v\rightarrow \alpha (1-\alpha )/2$ obtained when $u\searrow 1/\alpha$ in the region in which $D=0$. This implies non-differentiability of the front when reaching the population threshold at which diffusion vanishes. Despite the above technicality, the overall shape of the wavefront *u*(*z*) hardly varies with $\alpha$, and is always close to the analytic result for $\alpha \rightarrow \infty$. This limit gives the classic account of a pulled wave - even diffusion active only at $u=0$ is enough to populate virgin territory. The state $u=0$ is unstable to population growth, so *f*(*u*) then provides an increase in population density up to the (stable) carrying capacity $u=1$. Repetition of these mechanisms pulls along the wave. As noted elsewhere (Canosa 1973) the width of the front, *L*, increases linearly with wavespeed, and therefore scales here with $\sqrt{D(0)}$. The wave profile (Fig. 2b) is changed only slightly by the value of $\alpha$.

Similar results are found for more-or-less any plausible diffusion function with *D*(0) sufficiently large. Figure 1 illustrates this for a somewhat arbitrarily chosen diffusion function *D*(*u*); the speed of the front is the same as for constant diffusion ($D=D(0)=0.5$ in this figure). We will return to the question what constitutes “sufficiently large”, but it is certainly true that all *D*(*u*) with a maximum at low density will move at the linear speed $c_L$. To see this, note that one may replace *D*(*u*) by $\max _uD(u)$ in the integrand of (12) to obtain an upper bound on speed which is equal to the linear lower bound in the case $\max _uD(u)=D(0)$.

Given that in all these cases the travelling waves are clearly pulled, and therefore governed by behaviour at low density, it is perhaps trite to point out that any behavioural mechanism that elevates mean-squared displacement (and hence diffusion) at low density over its values at other densities, would lead in this model to elevated speeds of species invasion or population expansion. We are not aware of this point having been made before; perhaps because the diffusion functions $D=u^m$ most frequently considered have only the pathological values $D=0$ or $D\rightarrow \infty$ at low density, depending on the sign of *m*. For calculations using constant diffusion, there was never a need to be explicit that diffusion at low density is key. Furthermore, in light of the linear stability analysis outlined in Sect. 2.1, it is perhaps not surprising that diffusion functions with $D\rightarrow \infty$ as $u\rightarrow 0$ produce unusual (accelerating) wave solutions (King et al. 2003).

### Positive Density Dependence Over a Baseline

**Fig. 3:**
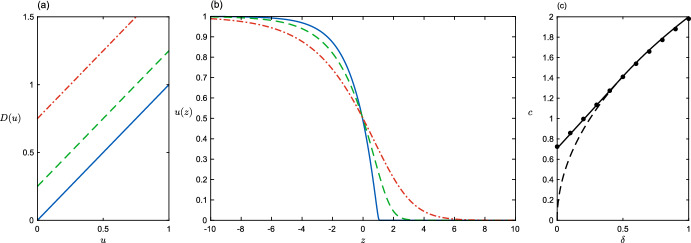
Positive density dependence over a baseline, $D(u)=u+\delta$ (**a**), with $\delta =0$ (the well-known degenerate diffusion case - solid line), $\delta =0.25$ (dashed line), $\delta =0.75$ (dot-dashed line). **b** The corresponding wavefronts *u*(*z*). **c** Wavespeed as a function of $\delta$. Dots are from simulation, solid line is theory (18), dashed line is the linear speed prediction $c_L$ (Color figure online)

In this section we consider the behaviour of travelling waves with logistic population growth $f(u) = u(1-u)$ and density dependent diffusion $D(u) = u + \delta$ where $\delta =D(0) \ge 0$ (Fig. 3a). Our first example of positive density-dependence, such diffusion functions might correspond to a population response to overcrowding, but with a background level of diffusion at all densities. Positive density-dependent dispersal of an invasive species into new territory, crayfish for example (Galib et al. 2022), could be suitably modelled by the PDE:

$$ \frac{\partial u}{\partial t}= \frac{\partial }{\partial x}\left[ (u+\delta )\frac{\partial u}{\partial x}\right] + u(1-u)\,. $$

The presence of the density-dependent term in the diffusion does not prohibit the linear analysis (for $\delta >0$), which gives the linear wavespeed $c_L=2\sqrt{\delta }$. When $\delta =0$ we have the sharp-fronted solution presented in the Introduction, with $c=1/\sqrt{2}$. Here we will reconcile these results; depending on the value of $\delta$, $c_L$ is either the selected speed, or it is not realisable.

To make use of the variational method (Sect. 2.2) in practice, it is necessary to identify appropriate trial functions *s*(*u*). Ideally there will be a family of functions characterised by a small number of parameters, so that the functional extremisation becomes algebraic. In the present case, we can make use again of the family $s(u)=u^{\beta }(1-u)^{-\beta }$ introduced above (15). In this way the infinite dimensional search of (12) is reduced to maximising over a single parameter. Completing the integrals, each of which can be written as a Beta function, yields the following bound:

18 $$\begin{aligned} \frac{1}{2}c^2 \ge \sup _{\beta \in [0,2)} \frac{1}{4}\beta (2-\beta +4\delta )={\left\{ \begin{array}{ll} \displaystyle \frac{(1+2\delta )^2}{4}\quad & \text {if}\quad \delta < 1/2,\\ 2\delta & \text {otherwise.} \end{array}\right. } \end{aligned}$$

In the region $\delta \ge \frac{1}{2}$, the bound on the speed matches the linear prediction, and is confirmed by numerical simulations (see Fig. 3c). More interestingly, when $\delta <\frac{1}{2}$, we have $c\ge (1+2\delta )/\sqrt{2}$, which exceeds the linear prediction. The switch occurs because below $\delta =\frac{1}{2}$ the maximum occurs somewhere in range of allowed $\beta$; beyond this, the maximum is always achieved in the upper limit, $\beta \nearrow 2.$

To show that this bound is tight we must check it is physically realisable. For the present choices of *D* and *f*, equation (6) becomes

19 $$\begin{aligned} v\big ((u+\delta )v'+v-c\big )+u(1-u)=0\,. \end{aligned}$$

Using our candidate *s* and *c* in equation (13), we obtain

$$ v=\frac{cs}{s'(u+\delta )}=\frac{u(1-u)}{\sqrt{2}(u+\delta )}\,, $$

which by inspection is a non-negative solution to (19) with the desired boundary conditions. Note the close parallel with the known solution for the so-called Nagumo equation mentioned in the Introduction. There $D=1$ and the reaction term $u(1-u)(u - a)$ for $0< a < 1/2$. Apart from the change of sign $(\delta =-a)$, the product of our $D=u+\delta$ and $f=u(1-u)$ is the Nagumo reaction term. The existence of this solution completes the proof that $c=(1+2\delta )/\sqrt{2}$ is the selected speed in this region, since it corresponds to the slowest realisable wave.

With access to the exact form of *v*, it is also possible to compute the shape of the wavefront when $\delta <\frac{1}{2}$. In the moving frame of reference $z=x-ct$ we have

$$ \frac{\text {d} z}{\text {d} u}=-\frac{1}{v}=-\frac{\sqrt{2}(u+\delta )}{u(1-u)}\,. $$

Integrating, and choosing $u(0)=\frac{1}{2}$, we obtain

20 $$\begin{aligned} z=\sqrt{2}\Big ((1+\delta )\log (1-u)-\delta \log (u)+\log (2)\Big )\,. \end{aligned}$$

Figure 3b shows a selection of these wavefronts. (20) can only be explicitly inverted for special values of $\delta$, including the limiting case $\delta =0$, where we obtain the known sharp-fronted solution $u(z)=\max \{0,1-\frac{1}{2}\exp (z/\sqrt{2})\}$. Finally, note that for the example with $\delta =0.25$ the front has the appearance of a pulled wave, with a tail that might induce a (false) prediction that its wavespeed should be the linear speed $c_L=2\sqrt{\delta }.$ These wavefront solutions are consistent with the smooth wavefronts reported by Sherratt (2010), who added a perturbation $\epsilon$ to the wavespeed $c^*=1/\sqrt{2}$. Since speed is proportional to $\delta$ for $\delta <0$, Sherratt’s $\epsilon =\sqrt{2}\delta$.

### Positive Density Dependence Above a Threshold

**Fig. 4:**
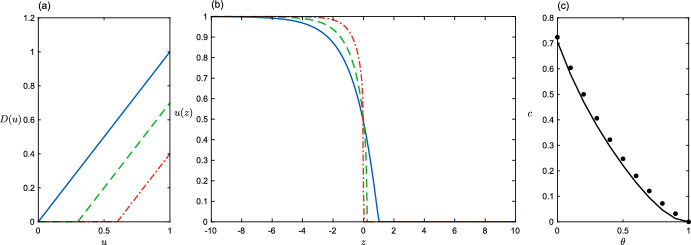
Positive density dependence above a threshold $D(u)=\max \{0, u -\theta \}$ (**a**), with with $\theta =0$ (the degenerate diffusion case - solid line), $\theta =0.3$ (dashed line), $\theta =0.6$ (dot-dashed line). **b** The corresponding wavefronts *u*(*z*) from simulation. **c** Wavespeed as a function of $\theta$. Dots are from simulation, solid line is the approximation (24) from theory (18) (Color figure online)

Finally, we will consider the diffusion function $D(u)=\max \{0, u -\theta \}$, $\theta \in [0,1)$ (Fig. 4a). We again have diffusion that increases linearly with density, but only now once the density has exceeded some critical value $\theta$. Biologically we have in mind that populations respond to overcrowding, but only once a threshold has been breached. An example of this behaviour can be seen in planthoppers (Denno and Roderick 1992) who, at low population densities produce flightless morphs. In dense populations, however, planthoppers produce fully winged forms with the ability to disperse away from the overcrowded population. Mathematically this case is of interest because prohibiting diffusion at small *u* certainly precludes pulled wavefronts with a small-*u* tail propagating at linear speed $c_L$. Figure 4 shows examples of *D*(*u*), *u*(*z*) for this case, and how wavespeed *c* varies with threshold $\theta$.

In this case, the family of trial functions $s=u^\beta (1-u)^{-\beta }$ used previously (15) results in a complicated expression for the bound on *c*, from which the supremum in $\beta$ cannot easily be taken. This could, in fact, be expected to be the case for most choices of diffusion function, since there is no a priori reason to expect that the integrals in (12) should have simple analytical forms. Instead, we shall pursue an alternative route to estimating the wavespeed, by directly approximating the maximal trial function $\hat{s}(u)$ which solves the ODE (6). To do this in turn requires knowledge (or approximation) of *v*(*u*), although in the present case it will in fact be easier to work with $w =D v$, which satisfies

21 $$\begin{aligned} w(w' - c) + f D = 0. \end{aligned}$$

**Fig. 5:**
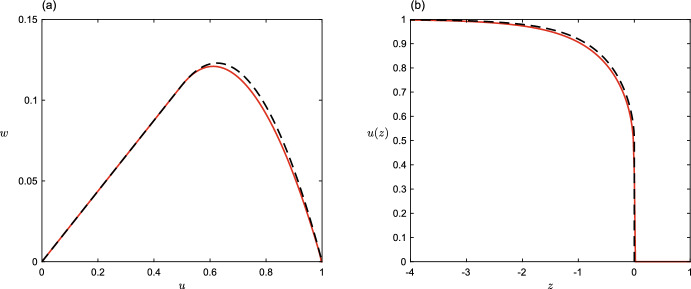
Comparison between numerical solution (solid line) and approximations (dashed line) for $w=Dv$ (**a**) and front shape (**b**), in the case $\theta =1/2$ (Color figure online)

Next we write down an approximation to the trajectory *w*(*u*), and use this to generate a trial function $\tilde{s}(u)$ which we take as an estimate of $\hat{s}(u)$ in the variational scheme, by using (14). In the range $u \in [0,\theta ]$, $D(u)=0$ so we simply have $w = cu$. In the range $u\in [\theta ,1]$ we approximate $w \approx c(1-u)(u-\theta ^2)/(1-\theta )^2$; this is the quadratic that yields continuity of *w* and its slope at $u=\theta$ and satisfies the condition $w(1)=0$ forced by $v(1)=0$ (see Fig. 5a). Substitution of *w* into (14) gives

22 $$\begin{aligned} \tilde{s}(u)=\exp \int \frac{c}{w} du \approx {\left\{ \begin{array}{ll} u\,\theta ^{\frac{1-\theta }{1+\theta }-1}& \text {if } u \le \theta \\ \displaystyle \left( \frac{u-\theta ^2}{1-u}\right) ^{\frac{1-\theta }{1+\theta }} & {\text {otherwise.}} \end{array}\right. } \end{aligned}$$

The resulting expression for the lower bound on wave speed is complicated. With some help from Mathematica, it may be written:

$$\begin{aligned} \begin{aligned} \frac{1}{2}c^2\ge \frac{(\theta +1) \left( \theta ^2-1\right) ^{\frac{\theta -1}{\theta +1}} \left( \theta ^3 \, H_2(\theta )-\left( 2 \theta ^2+\theta -1\right) \theta \, H_1(\theta )+(\theta -1) (\theta +1)^2 \, H_3(\theta )\right) }{(\theta -1)^{-\frac{1-\theta }{1+\theta }-3}\theta ^{2-\frac{1-\theta }{1+\theta }} (\theta +3)-(\theta -1)^{-3} (\theta +1) \left( \theta ^2-1\right) ^{1-\frac{1- \theta }{1+\theta }} \, H_1(\theta )} \end{aligned} \end{aligned}$$

where

23 $$\begin{aligned} \begin{aligned}&H_1(\theta )=\, _2F_1\left( -\frac{2 \theta }{\theta +1},\frac{\theta +3}{\theta +1};2+\frac{2}{\theta +1};\frac{1}{\theta +1}\right) \\&H_2(\theta )=\, _2F_1\left( \frac{\theta +3}{\theta +1},\frac{2}{\theta +1}-1;2+\frac{2}{\theta +1};\frac{1}{\theta +1}\right) \\&H_3(\theta )=\, _2F_1\left( \frac{\theta +3}{\theta +1},\frac{2}{\theta +1}-3;2+\frac{2}{\theta +1};\frac{1}{\theta +1}\right) \,,\\ \end{aligned} \end{aligned}$$

and $_2F_1$ is the hypergeometric function. The bound is, though, very well approximated (see Fig. 4c) by the simple expression

24 $$\begin{aligned} c\approx \frac{1}{\sqrt{2}}(1+\theta ^2)(1-\theta )^2\,. \end{aligned}$$

Perhaps not surprisingly, the travelling waves are sharp-fronted for all values of $\theta$. Using our approximations for *w* and *c* we find a close approximation to the inverse of the wavefront, centred at zero (see Fig. 5b):

25 $$\begin{aligned} z=-\int \frac{D}{w}\text {d}u \approx {\left\{ \begin{array}{ll} \frac{\sqrt{2}}{(1+\theta ) \left( 1+\theta ^2\right) } \left( \theta \log \left( \frac{u-\theta ^2}{\theta -\theta ^2 }\right) +\log \left( \frac{1-u}{1-\theta }\right) \right) \quad & u>\theta \\ 0& \text {otherwise.} \end{array}\right. } \end{aligned}$$

**Table 1:** Summary of the different types of diffusion explored in the paper, all with logistic growth $f(u) = u(1-u)$ with growth rate $r=1$

Density dependence	Wavespeed	Shape of wave
None, constant *D* (Fisher 1937; Kolmogorov et al. 1937)	$c=2\sqrt{D}$	Smooth
Positive, $D=u$ (Aronson 1980; Newman 1980)	$c=1/\sqrt{2}$	Sharp-fronted
Negative, unbounded at zero $D(u)=u^{m},\,\,m<0$ (King et al. 2003)	$c\rightarrow \infty$	No permanent solution
Negative, piecewise linear $D(u)=\max \{0,1-\alpha u\},\,\,\alpha >0$ (Sect. 3.1)	$c=2\sqrt{D(0)}$	Continuous everywhere, not differentiable at $u=1/\alpha$
Positive linear over a baseline $D(u)=u + \delta , \,\,\delta > 0$ (Sect. 3.2)	$c=\max \{2\sqrt{\delta },(1+2\delta )/\sqrt{2}\}$	Smooth
Positive linear with a threshold $D(u)=\max \{0, u - \theta \},\,\, \theta > 0$ (Sect. 3.3)	$c\approx \frac{1}{\sqrt{2}}(1+\theta ^2)(1-\theta )^2$	Sharp-fronted, discontinuous at $u=\theta$

The speed and a description of the shape of the travelling wave solution is given in each case

As in the other cases considered, the width of the front decreases with wavespeed.

## Discussion

In light of the increasing empirical attention being given to large-scale animal movement, and in recognition of how that movement will often depend on population density, our motivation for this paper was to put the density function on a more similar footing to the growth function in PDE models of population invasion. To that end, we have explored travelling wave solutions of the density-dependent FKPP equation (2) for a broader range of density functions *D*(*u*) than has previously been considered. A summary of our results, the speed and shape of travelling waves for a range of diffusion functions *D*(*u*) is given in Table 1. Travelling waves propagate at the minimum realisable speed *c*. For most choices of *D*(*u*), $c=c_L$, the (minimum) linear speed determined by the value of *D*(*u*) and the derivative of the growth term *f*(*u*) as $u\rightarrow 0$. The shape of the (pulled) front is affected very little by the details of *D*(*u*) for $u>0$. Most of the key properties of solutions to (2) can therefore be found through analysis of linear diffusion functions. The key condition needed for $c_L$ to be the selected speed is that *D*(0) is sufficiently greater than zero, but finite. A consequence of the dependence of *c* on *D*(0) is that any behavioural mechanism that increases diffusion at low density could in principle have a strong effect on the speed of population dispersal. For example, if the population consists of two types, chasers and escapees — as considered in the context of pattern formation by Surendran et al. (2019) and others — one could imagine that a chaser locked onto a single escapee might move further (at low density of escapees) than one that was switching between a choice of targets.

If *D*(0) is small or zero, variational methods allow us to compute minimum realisable speeds, enabling us to extend known results for $D(u)=u$ to diffusion functions with positive density dependence over a baseline $\delta$ (Sect. 3.2) and above a threshold $\theta$ (Sect. 3.3). For the former, $c>c_L$ up to a critical value of the baseline $\delta$; wave profiles are smooth for $\delta >0$. All wave profiles in the threshold case are sharp-fronted, and the selected speeds decrease monotonically with $\theta$.

The variational principle provides a promising method of estimating the speed of travelling waves in cases where exact calculation is impossible. Care must be taken when selecting the trial function *s*(*u*) to ensure the integrals in (7) exist. This is often non-trivial or, as we have seen in Sect. 3.3, can sometimes only be approximated. We note also that our choices of functions *D*(*u*) (made to be of the simplest linear forms, consistent with being able to capture the fundamental behaviour of interest) has led us to some mathematical difficulties when the value of the diffusion function becomes zero, or when required to differentiate a non-smooth function. We would expect that any real system will be modelled with a smooth *D*(*u*), eliminating any issues around differentiability. It also seems likely that routine, background levels of animal movements — such as for foraging and mate-searching — might produce non-zero diffusion at all densities in many cases. Future work should make further progress in bridging the gap between mathematical models and population dispersal. An obvious next step would be to model populations of two types of individuals, such as the chasers and escapees considered by Surendran et al. (2019); other likely candidates for ‘types’ are the sexes, as males and females have been found to exhibit different density dependent dispersal in various wild populations eg. Fattebert et al. (2015); Kopsala et al. (2023). A coupled system of PDEs with different *D*(*u*) within- and between-sex would hence be a interesting focus of future work.

## Data Availability

This manuscript has no associated data.
